# Vitamin D Status and Quality of Life in Healthy Male High-Tech Employees

**DOI:** 10.3390/nu8060366

**Published:** 2016-06-15

**Authors:** Sigal Tepper, Yael Dabush, Danit R. Shahar, Ronit Endevelt, Diklah Geva, Sofia Ish-Shalom

**Affiliations:** 1 The S. Daniel Abraham International Center for Health and Disease, Department of Public Health, Faculty of Health Sciences, Ben-Gurion University, Beer-Sheva 8410501, Israel; dshahar@bgu.ac.il (D.R.S.); diklah.geva@gmail.com (D.G.); 2 Department of Clinical Nutrition, Rambam Health Care Campus, Haifa 31093, Israel; yaelidabush@gmail.com; 3 School of Public Health, Haifa University, Mt. Carmel, Haifa 31905, Israel; ronit.endevelt@moh.gov.il; 4 Department of Endocrinology & Diabetes, Elisha Medical Center, Haifa 3463626, Israel; sishshalom@gmail.com

**Keywords:** quality of life, vitamin D, 25(OH)D, healthy men

## Abstract

While low vitamin D status has been shown to be associated with decreased quality of life in unhealthy populations and women, only limited data are available regarding healthy adult men. Our aim was to evaluate the associations between health-related quality of life (QoL) and vitamin D status in adult men. High-tech employees aged 25–65 year were recruited from an occupational periodic examination clinic at Rambam Health Campus. QoL was assessed using the Centers for Disease Control and Prevention (CDC) Health-related quality of life questionnaire (HRQOL-4). Serum 25-hydroxyvitamin D (25(OH)D) and Body Mass Index (BMI) were measured; further information was collected about physical activity, education, sun exposure, sick-days, and musculoskeletal pain severity (visual analog scale). Three hundred and fifty-eight men were enrolled in the study; mean serum 25(OH)D level was 22.1 ± 7.9 ng/mL (range 4.6–54.5 ng/mL). In a multivariate logistic regression model, 25(OH)D was a significant independent determinant of self-rated health; Odds Ratio (OR) for self-rated health was 0.91 (95% confidence interval (CI) 0.85–0.97, *p* = 0.004), adjusted for age, BMI, pain severity, physical activity, and sun exposure. Every 1 ng/mL increase of 25(OH)D was associated with 9% reduction in the odds of reporting self-rated health as fair or poor. Poisson regression model demonstrated an association between physically unhealthy days and 25(OH)D levels (rate ratio 0.95, *p* < 0.001). In conclusion, serum levels of 25(OH)D were associated with self-rated health and with physically unhealthy days of HRQOL in healthy high-tech male workers. Future intervention studies are required to test the impact of vitamin D supplementation on QoL.

## 1. Introduction

There is growing evidence that vitamin D plays an important role in a range of physiological and pathological processes involved in bone health, physical functioning, muscle strength, various chronic diseases [1,2,3] and, as recently shown, depressive disorders [4,5]. Accumulating evidence over the past decade reveals a high prevalence of vitamin D deficiency and insufficiency with numbers ranging from 20% to 100% depending on the population and definition of deficiency [6,7]. In a healthy population deficiency might be related to modern lifestyles, which are typified by long work hours indoors and inadequate mid-day sun exposure (required for the body to produce vitamin D), sometimes co-occurring with the use of protective measures to avoid overexposure to the sun outside working hours [8,9].

Quality of life (QoL) is a subjective evaluation of physical and mental functioning influenced by age, gender, employment status, marital status, and education [10,11]. Recent studies suggest that there is a positive association between vitamin D status and QoL although data is inconsistent due to differences in studied populations and QoL assessment tools [12,13,14,15,16]. Some studies have suggested that other factors, such as depression [17,18], chronic diseases [19], and pain [20,21], affect the Qol score. Perhaps the vitamin D effect on QoL is partly expressed through these factors, which are part of the QoL.

The association between vitamin D status and QoL was tested among various populations including elderly subjects [22,23], unhealthy populations of various ages [14,15,24], and on post-menopausal women [13,15,16,24]. Intervention studies are even more inconsistent: while vitamin D supplementation did not improve QoL in elderly heart failure patients [25], among community-dwelling elderly [26] and veterans with chronic pain [27], vitamin D supplementation demonstrated improvement in QoL. The improvement was manifested in reduced pain [26,27], better sleep quality [27], and better functional mobility [26]. Although recent findings revealed high rates of vitamin D insufficiency in healthy young and middle-aged men, they are still an understudied population and most data are from studies in women. The association between levels of vitamin D and QoL has not been tested in healthy men. The objective of the present study is to assess the association between Health-related quality of life questionnaire (HRQOL) and vitamin D status among healthy adult high-tech workers. We hypothesize that serum 25-hydroxyvitamin D (25(OH)D) levels are predictors of HRQOL components, independent of other factors that have been found associated with QoL, such as Body Mass Index (BMI), pain, sun exposure, and physical activity.

## 2. Experimental Section

### 2.1. Study Population and Study Design

High-tech employees were recruited between October 2009 and September 2010 on the occasion of their annual examination at the Rambam Health Care Campus clinic in Haifa, Israel, and via the study’s website. The study was approved and monitored by the human subjects ethics committee of Rambam Health Care Campus (HL-3153). Each participant provided written informed consent at the beginning of the study. Eligible participants were men aged 25−65 who, according to their medical records, self-reported questionnaires, and laboratory evaluations, did not suffer from any active severe condition and whose laboratory results revealed no significant abnormalities. Anthropometric and laboratory data were collected by the clinic nurses. Demographic, QoL, and lifestyle questionnaires were sent to the participants by e-mail on the same day.

### 2.2. Serum 25(OH)D

A blood sample was drawn by venipuncture after a 12-h fast. Serum 25(OH)D was measured by chemiluminescent immunoassay (DiaSorin, Inc., Stillwater, MN, USA) at the Rambam Health Care Campus laboratory. The functional sensitivity of this test was <4.0 ng/mL. Intra- and inter-assay coefficients of variation (CV) were between 6.7% and 8.6% for the range of 17−60 ng/mL 25(OH)D. For external validation, the laboratory participated in the vitamin D External Quality Assessment Scheme [28] and the values tested were within the acceptable range.

### 2.3. Measurements

Anthropometric—Weight in kg and height in cm (Shekel Healthweigh, Model H141, Beit Keshet, Israel) were measured while participants were barefoot and dressed in light clothing. BMI was calculated as weight in kilograms divided by height in meters squared (weight (kg)/height (m^2^)).

Questionnaires—Participants’ personal and occupational lifestyle data and medical data, including lists of medications and dietary supplements, were collected with appropriate questionnaires sent via e-mail. Musculoskeletal pain severity was assessed using a visual analog scale (VAS). Non-responders were prompted after one week via e-mail and, thereafter, they were reminded weekly by e-mail and phone calls.

Quality of life: QoL was determined using the Centers for Disease Control and Prevention (CDC) health-related quality of life questionnaire (HRQOL-4). HRQOL-4 uses four questions called “Unhealthy Days Measure” relating to the participants’ health-related quality of life over the 30-day period before the scheduled visit. High and consistent correlations of CDC HRQOL measurements with other Quality of Life measurements, such as the SF-36, in a general community and a population of persons with known disabilities, have confirmed its good validity [29,30]. The four component questions were: (1): rate your health status as poor, fair, good, very good, or excellent; (2) thinking about your physical health, which includes physical illness and injury, for how many days during the past 30 days was your physical health not good; (3) thinking about your mental health, which includes stress, depression, and problems with emotions, for how many days during the past 30 days was your mental health not good; and (4) about how many days did poor physical or mental health keep you from doing your usual activities, such as self-care, work, or recreation.

Physical activity—A questionnaire adapted for use in the current study was used to calculate physical activity [31,32]. The questionnaire included information on type and frequency of various activities, including aerobic/anaerobic activities.

Sun exposure—Sun exposure was determined using a validated questionnaire [33].

### 2.4. Statistical Methods

For analysis, the "unhealthy days" count (the number of unhealthy days during the past 30 days experienced by the participants) has been used [34]. Self-rated health was collapsed to two levels: excellent/very good/good and fair/poor. The physically unhealthy days, mentally unhealthy days, and activity limitation days (components 2–4) were collapsed into three levels: 0; 1–7; and ≥8 days. To test the associations between study variables and QoL, HRQOL components was tabulated according to 25(OH)D level and tested with chi-square. Results are given for the cutpoints following Institute Of Medicine (IOM) convention [35] (similar trends were seen following different 25(OH)D cutpoints). Univariate and multivariate logistic regression models were applied to estimate the association between self-rated health and 25(OH)D and different determinants of QoL (age, BMI, pain severity, physical activity, sun exposure, marital status, and education). We used a *P* value for entry set at 0.15 and that for staying at 0.20 to select the predictors in the multivariate models, where self-rated health was the dependent variable. Multivariate analysis was carried out using Poisson regression with the count of the number of Unhealthy days (physically, mentally, activity limitation, and summary of all three components of unhealthy days) as the outcome. This model was run first for 25(OH)D alone and then for the 25(OH)D adjusted for cofactors shown to be significant (age, BMI, pain severity, physical activity, and sun exposure). Negative-binomial regression, which accounts for overdispersion, showed similar results as the Poisson regression. Reported here are the rate ratios from the Poisson regression. All analyses were conducted using SPSS version 23 statistical software (IBM corp, Armonk, NY, USA).

## 3. Results

Out of 443 men assessed for eligibility, 25 men did not meet the inclusion criteria and 60 men declined to participate; thus, the participation rate was 86%. 358 men completed physical examinations, lab assessments, and questionnaires. Out of 358 participants, four did not complete the questionnaires and were excluded from the QoL analysis.

The mean serum 25(OH)D level was 22.1 ± 7.9 ng/mL (range 4.6–54.5 ng/mL), 93% of the participants reported excellent/very good or good health status, 66% reported zero physically unhealthy days, 69.7% reported zero mental unhealthy days, and 84.3% reported zero days of activity limitation.

For descriptive analysis, serum 25(OH)D levels were divided into three categories in line with IOM recommendations (≤12 ng/mL, 12–20 ng/mL, ≥20 ng/mL) [35]. Baseline characteristics according to the IOM 25(OH)D categories are presented in Table 1. A significant difference across 25(OH)D levels was shown for BMI only (*p* = 0.019).

To illustrate the association of HRQOL 4 components across 25(OH)D categories we collapsed the number of days into three categories: none (0), up to one week (1–7 days), and more than a week (>7 days), Results are shown in Table 2. Better self-rated health was associated with a higher category of 25(OH)D levels: 86.6% in 25(OH)D ≤12 ng/mL, 89.1% in 12 < 25(OH)D ≥ 20 ng/mL, and 96.2% >20 ng/mL (*p* = 0.019). In contrast, for physically- and mentally-unhealthy days, no considerable difference distributions have been found.

The logistic regression model of self-rated health as the dependent variable shown in Table 3 demonstrates that 25(OH)D is independently associated with self-rated health, after adjusting for age, BMI, pain, physical activity, and sun exposure (*p* = 0.026). Every 1 ng/mL increment of 25(OH)D was associated with 9% reduction in the odds of reporting self-rated health as fair or poor.

Multivariate analysis was carried out using Poisson regression with the count of the number of unhealthy days reported for each component (physical, mental, activity limitation, and total unhealthy days). Reported here are the rate ratios from the Poisson regression, crude and after adjustment for age, BMI, pain, physical activity, and sun exposure (Table 4). Every 1 ng/mL increment of 25(OH)D was associated with 4% reduction in the rate of physically unhealthy days. Activity limitation and mentally-unhealthy days were not associated with 25(OH)D.

## 4. Discussion

In this study among 356 healthy high-tech employees at high risk of vitamin D deficiency or insufficiency we showed an association between health-related quality of life and vitamin D status. We demonstrated positive associations between self-rated health and serum 25(OH)D level and negative association between physically unhealthy days and serum 25(OH)D levels. Participants with higher levels of vitamin D had fewer unhealthy days and reported better self-rated health.

Data on the relationship between vitamin D status and QoL are conflicting. A survey of post-menopausal women found no association between vitamin D deficiency and QoL [16], while another study in pre-menopausal healthy women showed that a lower vitamin D status was associated with lower QoL [15]. A positive association between QoL and vitamin D status has, likewise, been reported in other studies; however, most of the studies were performed among known susceptible populations, such as the elderly or among depressed, obese, and otherwise unhealthy individuals [13,24,25,26,36,37,38].

A recent systematic review of intervention studies found that overall, vitamin D supplementation was not associated with significant changes in quality of life. The authors did find small to moderate effect of vitamin D on quality of life when used on a short-term basis in diseased populations. However, only three studies were conducted in healthy/non-diseased populations, all of them in women, mostly elderly, and with low dose supplementation. Therefore, more studies are needed in young and middle-aged men [39].

We found an increased rate of physically unhealthy days for participants with lower 25(OH)D levels. The positive association between vitamin D status and physical functioning in elderly populations is well documented [25,40,41,42]. As far as we know, this is the first study to identify a similar relationship among healthy young and middle-aged adults. Additionally, we found that an increase in 25(OH)D levels was associated with higher reported self-rated health. Self-rated health has been found to predict sickness absence [43,44] and, in long-term follow-ups, self-rated health had a predictive validity in relation to morbidity and mortality [43,45]. Considering that vitamin D deficiency and insufficiency treatment is simple and reasonably priced, it may be beneficial to test its efficacy in intervention studies in decreasing sick days and improving QoL in young and middle-aged men.

Since vitamin D affects many physiological domains, there are several ways in which it can alter health and physical wellbeing: 1. Vitamin D acts as a potent modulator of immune responses, both innate and adaptive. Therefore, vitamin D insufficiency or deficiency may impair immune responses and predispose individuals to infection. Indeed, low serum concentrations of 25(OH)D have been linked to infectious diseases. In addition, vitamin D affects several pathways related to regulation of inflammatory response [46]. 2. Vitamin D has a role in muscle function. Vitamin D deficiency may account for muscle weakness and musculoskeletal pain, and its level is strongly correlated with muscle quality and lower muscle fat infiltration. Lately it has been suggested that vitamin D also has a role in energetics; a recent study demonstrated that vitamin D level correlates with maximal oxygen consumption and that supplementation of vitamin D increases fitness in athletes. 3. Vitamin D receptors are found in high concentration in various areas of the brain. Low vitamin D levels have been associated with cognitive decline and mood disorders [46].

Our study suffers from some limitations that need to be addressed. Since we chose to focus on a specific segment of the adult population, *i.e.*, healthy adult males, who are known to have better QoL compared with the general population, we cannot generalize our results to all adult populations. Additionally, our study focused on a homogeneous population characterized by high socioeconomic status and good health. Most of the participants were aware of the importance of a healthy lifestyle. Indeed, a comparison with the general population revealed differences in QoL scores as well as higher education level in the study’s participants [47]. Given the homogeneity of the population in our study, major determinants of quality of life such as gender, education, marital status, and employment status could not be tested. We used the Diasorin assay, which has raised some concerns in the past with regard to an underestimation compared to liquid chromatography-tandem mass spectrometry (LC-MS/MS—usually selected as the nominal gold standard). Therefore, an underestimation of 25(OH)D levels cannot be entirely ruled out.

In summary, in healthy men with a high QoL score, serum 25(OH)D levels were associated with better self-rated health and physical health in the past 30 days. It will be interesting to further explore these relationships in more heterogeneous populations. Future intervention studies are required to test the impact of vitamin D supplementation on QoL.

## Figures and Tables

**Table 1:** Characteristics of the study population across 25(OH)D (ng/mL) levels.

Characteristic	≤12 ng/mL	12–20 ng/mL	>20 ng/mL	Total	*P* **
	(*n* = 38)	(*n* = 110)	(*n* = 210)	(*n* = 358)	
Age (years)	49.57 ± 11.06	49.8 ± 10.63	48.13 ± 9.81	48.8 ± 10.21	0.34
BMI (kg/m^2^)	28.53 ± 4.45	27.05 ± 3.71	26.61 ± 3.67	26.97 ± 3.81	0.019
Physical Activity (h/week)	1.58 ± 1.96	2.36 ± 2.9	2.57 ± 2.67	2.4 ± 2.69	0.111
Sun Exposure (h/week)	0.89 ± 0.63	0.94 ± 0.54	0.98 ± 0.51	0.96 ± 0.53	0.583
Sick days (per year)	2.87 ± 3.46	2.29 ± 3.45	2.5 ± 4.91	2.47 ± 4.36	0.77
Musculoskeletal Pain (% yes, (*n*))	53 (20)	47 (52)	54 (113)	52 (185)	0.437
Pain Severity ^§^ (VAS)	1.53 ± 1.77	1.4 ± 1.9	1.57 ± 1.83	1.51 ± 1.84	0.738
Education (% Academic, (*n*))	76 (29)	89 (98)	88 (184)	87 (311)	0.082

**Table 2:** HRQOL-4 components across 25(OH)D (ng/mL) levels.

	≤12 ng/mL (*n* = 38)	12–20 ng/mL (*n* = 110)	≥20 ng/mL (*n* = 208)	Total (*n* = 356)	*P* value *
Self-rated health, % (*n*)					
excellent, very good, good	86.8% (33)	89.1% (98)	96.2% (200)	93% (331)	*0.019*
fair, poor	13.2% (5)	10.9% (12)	3.8% (8)	7% (25)	
Physical unhealthy days					
0	68.4% (26)	68.2% (75)	64.4% (134)	66.0% (235)	0.141
1–7	23.7% (9)	29.1% (32)	34.1% (71)	31.5% (112)	
8 and over	7.9% (3)	2.7% (3)	1.4% (3)	2.5% (9)	
Mental unhealthy days					
0	76.3% (29)	71.8% (79)	67.3% (140)	69.7% (248)	0.708
1–7	23.7% (9)	26.4% (29)	31.3% (65)	28.9% (103)	
8 and over	0% (0)	1.8% (2)	1.4% (3)	1.4% (5)	
Activity limitation days					
0	86.8% (33)	87.3% (96)	82.2% (171)	84.3% (300)	0.132
1–7	13.2% (5)	10.9% (12)	17.8% (37)	15.2% (54)	
8 and over	0% (0)	1.8% (2)	0% (0)	0.6% (2)	
Total unhealthy days ^§^					
0	57.9% (22)	50.9% (56)	48.1% (100)	50% (178)	
1–7	31.6% (12)	40.9% (45)	46.2% (96)	43% (153)	0.443
8 and over	10.5% (4)	8.2% (9)	5.8% (12)	7% (25)	

* chi-square test; ^§^ sum of physical and mental unhealthy days.

**Table 3:** The association between 25(OH)D (ng/mL) and self-rated health.

	Fair/Poor (*vs.* Excellent-good)	
	OR (95% CI)	*P* ^§^
Crude OR:		
25(OH)D (ng/mL)	0.91 (0.83–0.91)	*0.003*
Adjusted OR *		
25(OH)D	0.93 (0.87–0.97)	*0.026*
Age (years)	1.02 (0.98–1.07)	*0.350*
Bmi (kg/h^2^)	1.06 (0.95–1.18)	0.323
Pain severity (VAS)	1.27 (1.04–1.55)	*0.022*
Physical Activity (h/week)	0.74 (0.56–0.98)	*0.033*
Sun exposure (h/week)	0.68 (0.24–1.91)	0.460

* Adjusted for: age (years), BMI, pain severity (musculoskeletal pain severity, VAS), physical activity (h/week), sun exposure (h/week); ^§^ logistic regression *P*-value.

**Table 4:** The association between 25(OH)D (ng/mL) and unhealthy days.

	Physically Unhealthy Days		Mentally Unhealthy Days		Activity Limitation		Total Unhealthy Days ^§^	
	RR (95% CI)	*P* *	RR (95% CI)	*P* *	RR (95% CI)	*P* *	RR (95% CI)	*P* *
Crude Rate Ratio (RR)								
25(OH)D (ng/mL)	0.96 (0.94–0.97)	*<0.001*	1.01 (0.99–1.02)	0.147	0.99 (0.97–1.01)	0.330	0.98 (0.97–0.99)	*0.001*
Adjusted Rate Ratio (RR) ^β^								
25(OH)D (ng/mL)	0.95 (0.94–0.97)	*<0.001*	0.98 (0.98–1.03)	0.060	0.99 (0.97–1.02)	0.503	0.99 (0.98–0.99)	*0.014*
Age (years)	0.96 (0.95–0.97)	*<0.001*	0.99	0.225	0.96 (0.94–0.98)	<0.001	0.98 (1.03–1.07)	*<0.001*
Bmi (kg/h^2^)	1.06 (1.04–1.09)	*<0.001*	1.04 (1.01–1.07)	*0.008*	0.97 (0.92–1.02)	0.259	1.05 (1.03–1.07)	*<0.001*
Pain severity (VAS)	1.29 (1.23–1.35)	*<0.001*	1.07 (1.01–1.13)	*0.024*	1.32 (1.23–1.42)	*<0.001*	1.18 (1.14–1.22)	*<0.001*
Physical Activity (h/week)	1.08 (1.04–1.11)	*<0.001*	0.98 (0.94–1.03)	0.414	1.11 (1.05–1.17)	*<0.001*	1.03 (1–1.06)	0.060
Sun exposure (h/week)	1.21 (0.99–1.48)	0.070	1.13 (0.92–1.39)	0.235	0.48 (0.31–0.75)	*<0.001*	1.15 (1–1.33)	0.056

^§^ sum of physical and mental unhealthy days; ^β^ Adjusted for: age (years), BMI, pain severity (musculoskeletal pain severity, VAS), physical activity (h/week), sun exposure (h/week); * Poisson regression *P* value.
